# Environmental and Wastewater Treatment Applications of Stimulus-Responsive Hydrogels

**DOI:** 10.3390/gels11010072

**Published:** 2025-01-16

**Authors:** Anita Ioana Visan, Irina Negut

**Affiliations:** National Institute for Lasers, Plasma and Radiation Physics, 077125 Măgurele, Romania; anita.visan@inflpr.ro

**Keywords:** stimulus-responsive hydrogels, wastewater treatment, adsorption and photodegradation, hydrogel functionalization, sustainable water management

## Abstract

Stimulus-responsive hydrogels have emerged as versatile materials for environmental and wastewater treatment applications due to their ability to adapt to changing environmental conditions. This review highlights recent advances in the design, synthesis, and functionalization of such hydrogels, focusing on their environmental applications. Various synthesis techniques, including radical polymerization, grafting, and copolymerization, enable the development of hydrogels with tailored properties such as enhanced adsorption capacity, selectivity, and reusability. The incorporation of nanoparticles and bio-based polymers further improves their structural integrity and pollutant removal efficiency. Key mechanisms such as adsorption, ion exchange, and photodegradation are discussed, emphasizing their roles in removing heavy metals, dyes, and organic pollutants from wastewater. Additionally, this review presents the potential of hydrogels for oil–water separation, pathogen control, and future sustainability through integration into circular economy frameworks. The adaptability, cost-effectiveness, and eco-friendliness of these hydrogels make them promising candidates for large-scale environmental remediation.

## 1. Introduction

Hydrogels are three-dimensional polymeric networks that can absorb large amounts of water while maintaining their structure. They are classified as stimulus-responsive or “smart” materials due to their ability to undergo significant changes in their physical or chemical properties in response to external stimuli such as pH, temperature, light, electric fields, and ionic strength [1,2,3]. These responsive properties are particularly advantageous for environmental applications, where the dynamic nature of pollutants necessitates adaptable treatment solutions.

Stimulus-responsive hydrogels are characterized by their ability to undergo reversible changes in volume and solubility in response to environmental stimuli. This behavior is primarily due to the presence of hydrophilic functional groups within their polymeric structure, which interact with water and other solvents [4,5]. The incorporation of nanoparticles, such as TiO_2_ and graphene, into hydrogel matrices can enhance their mechanical strength and adsorption capacity, making them more effective for pollutant removal [6,7].

Hydrogels can be designed to swell or shrink based on the pH of the surrounding environment. For instance, pH-sensitive hydrogels can selectively adsorb heavy metal ions or organic dyes depending on the pH level, facilitating targeted pollutant removal [7,8].

Temperature-responsive hydrogels exhibit changes in their physical state at specific temperatures, known as the lower critical solution temperature (LCST). This property allows for the controlled release of adsorbed pollutants, making them suitable for dynamic wastewater treatment processes [3,9].

Light-responsive hydrogels can undergo photodegradation of organic contaminants when exposed to UV light, providing an energy-efficient method for pollutant removal [10,11]. The incorporation of photocatalytic materials within the hydrogel matrix can enhance this effect, allowing for the simultaneous adsorption and degradation of pollutants.

The application of electric fields can induce movement in charged hydrogels, enhancing their adsorption capabilities. Similarly, changes in ionic strength can affect the swelling behavior of hydrogels, influencing their interaction with pollutants [2,12].

The unique properties of stimulus-responsive hydrogels make them invaluable in environmental applications. Their ability to selectively adsorb pollutants, regenerate after use, and respond to environmental changes allows for more efficient and sustainable wastewater treatment solutions. For example, hydrogels have been successfully employed to remove organic dyes, heavy metals, and other contaminants from wastewater, demonstrating high adsorption capacities and rapid response times [13,14,15]. Furthermore, the integration of magnetic properties into hydrogels facilitates easy recovery and reuse after treatment, addressing one of the significant challenges in conventional wastewater treatment methods [16,17].

The responsive nature of hydrogels can be harnessed to create adaptive treatment systems that respond to varying pollutant concentrations and types in real time. This adaptability not only improves the efficiency of pollutant removal but also minimizes the environmental impact associated with traditional treatment methods, such as chemical flocculation and sedimentation [8,18]. By utilizing hydrogels that can respond to multiple stimuli, it is possible to develop multifunctional materials capable of addressing a wide range of environmental challenges, from heavy metal ion removal to organic pollutant degradation [6,19,20].

In the review by Md. Ahmaruzzaman et al., they discussed adsorption which stands out as one of the simplest techniques for purifying contaminated water, and they elucidated the primary interactions involved in the adsorption processes of water pollutants using hydrogel-based adsorbents, facilitating a better understanding of their removal mechanisms. The authors highlighted the preparation, characterization, and adsorption properties of hydrogels for water purification. Their review evaluated the benefits of these polymeric materials in removing various organic and inorganic contaminants [21].

Vibha Sinha et al. explained how polymeric networks can be modified to enhance their functional groups, resulting in a strong affinity for various contaminants found in wastewater. Their remarkable capacity to absorb large volumes of water makes them ideal candidates for smart materials in water remediation. Their work focuses on the latest developments in hydrogel modifications and types, particularly regarding their adsorption capabilities for metal ions and dyes, by making a comparative analysis of adsorption mechanisms, kinetics, isotherms, and relevant thermodynamic parameters of modified hydrogels [22].

Kumar et al. underlined how numerous technologies and materials have been developed for treating contaminated water, but many of these methods are expensive and can generate secondary pollutants. Researchers explained how hydrogels can remove impurities from water through mechanisms such as physio-adsorption, chemo-adsorption, sorption, and other secondary forces. Kumar and colleagues provide detailed insights into the toxicants present in water, the different types of hydrogels, and their synthesis techniques by assessing their effectiveness in removing specific toxicants from water. Additionally, current trends involve modifying hydrogels to enhance their functionality for specific applications, positioning these tailored hydrogels as emerging tools for targeted water purification efforts [23].

Enas et al. discussed the technologies used in hydrogel production, including process design implications, block diagrams, and optimized preparation conditions [24].

In a recent work, the authors examined the removal of contaminants from water utilizing hydrogel-based materials by emphasizing the recent findings, ongoing challenges, and potential advancements in the field. Polymer and graphene hydrogels have emerged as promising materials for eliminating both inorganic (such as metals and metalloids) and organic (such as dyes) trace contaminants from water. These hydrogels can be combined with various traditional or innovative adsorbents to enhance their removal efficiency. One of the key characteristics of hydrogels is their ability to form three-dimensional networks, which impart unique properties to these materials [25].

The development and application of stimulus-responsive hydrogels represent a promising frontier in environmental science and engineering. Their unique properties and capabilities can significantly enhance wastewater treatment processes, contributing to more sustainable and effective environmental management strategies.

The present work offers an in-depth examination of the role of stimulus-responsive hydrogels in mitigating environmental pollution, particularly in wastewater treatment. It begins by defining hydrogels as three-dimensional polymer networks that can absorb significant amounts of water and respond to various external stimuli, such as pH and temperature, making them ideal for adaptive environmental applications. This review discusses multiple synthesis methods, including radical polymerization and grafting, and emphasizes functionalization’s importance in improving their pollutant removal capabilities. We categorize hydrogels based on their origins, compositions, and responsiveness while also exploring pollutant removal mechanisms, such as adsorption and photodegradation. Furthermore, this review highlights various applications in wastewater treatment, detailing the efficacy of hydrogels in removing heavy metals, organic pollutants, and pathogens through illustrative case studies. Conclusively, it identifies future directions and challenges in the field, stressing the need for cost-effective production and regulatory compliance.

This review stands out from the other articles already published because its comprehensive approach to linking hydrogels’ specific properties and functionalization with their practical applications in environmental management offers insights not fully explored in previous studies, especially regarding the adaptive capabilities that enhance pollutant removal efficiency.

## 2. Synthesis and Functionalization of Stimulus-Responsive Hydrogels for Environmental Use

### 2.1. Methods of Hydrogel Synthesis

Although the literature is rich with reviews on hydrogel synthesis techniques, this subchapter aims only to summarize key advancements within each method, with a particular focus on their relevance to environmental and biomedical applications. Numerous reviews offer comprehensive insights into the principles and applications of these synthesis techniques.

**Figure 1:**
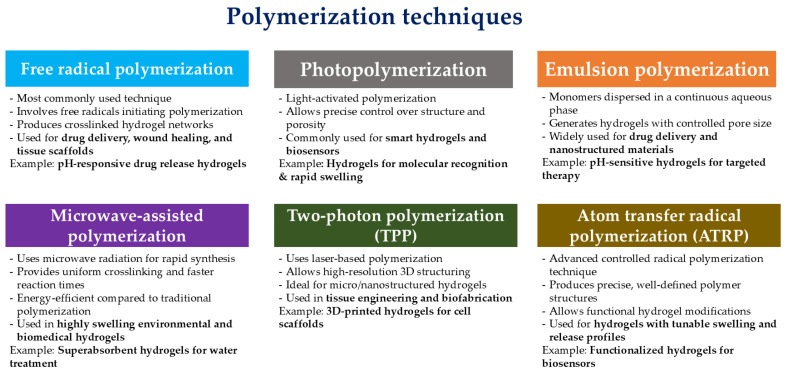
Polymerization techniques for obtaining hydrogels.

(i) Polymerization (Figure 1), particularly free-radical polymerization, remains one of the most common approaches for hydrogel formation, allowing for controlled network structures and tunable properties. This method is popular due to its efficiency and ability to produce homogenous hydrogels across various environments, including those that mimic biological conditions [26]. Techniques like atom transfer radical polymerization (ATRP) further refine this approach by enabling precise molecular weight control and narrow polymer distribution, contributing to advanced hydrogel applications in biomedical fields [27].

Free radical polymerization is one of the most widely used techniques for synthesizing hydrogels. This method involves the generation of free radicals that initiate the polymerization of monomers, leading to the formation of a crosslinked network. The process can be performed in various media, including aqueous solutions, which is particularly advantageous for biomedical applications. Suhail et al. demonstrated the use of free radical polymerization to create pH-responsive semi-interpenetrating polymer network hydrogels for controlled drug release. Their study highlighted that the polymerization technique resulted in enhanced thermal stability and mechanical properties due to effective grafting and cross-linking of the hydrogel components [28]. Similarly, Mittal et al. utilized free radical polymerization to develop hydrogels from poly(dimethylaminoethyl acrylate) and hyaluronic acid, showcasing the versatility of this technique in producing hydrogels with desirable properties for wound healing applications [29].

Atom transfer radical polymerization (ATRP) is a controlled radical polymerization technique that allows for the synthesis of well-defined polymer architectures. This method can be employed to create hydrogels with specific functionalities and properties. Zhang discussed the application of ATRP in synthesizing water-soluble chain transfer agents for gel dispersions, demonstrating the potential of this technique in producing hydrogels with controlled properties [30]. The ability to tailor the polymer structure through ATRP makes it a valuable approach in hydrogel synthesis.

Photopolymerization is a technique that employs light to initiate polymerization reactions. This method allows for precise control over the spatial and temporal aspects of hydrogel formation, making it suitable for applications that require specific patterns or structures. Nicol reviewed the use of photopolymerization in creating porous hydrogels, emphasizing its versatility and effectiveness in generating hydrogels with tailored porosity and mechanical properties [31]. Additionally, Kawamura highlighted the use of photopolymerization in synthesizing molecule-responsive hydrogels, which exhibited rapid swelling and shrinkage in response to target molecules, showcasing the potential of this technique for smart applications [32].

Copolymerization allows for the combination of multiple monomers into a single hydrogel network, imparting diverse functional groups and environmental responsiveness. This method enables the synthesis of multifunctional hydrogels, such as thermos-responsive hydrogels that exhibit reversible phase transitions, making them ideal for tissue engineering and drug delivery [33].

Another prominent method is emulsion polymerization, and it is particularly useful for producing hydrogels with a microporous structure. Emulsion polymerization allows for high control over particle size, resulting in hydrogels with a high surface area and enhanced absorption properties. This method is advantageous for applications in environmental and water treatment, where high pollutant absorption is needed, as well as in drug delivery systems that benefit from increased surface interaction and drug loading [34].

Microwave-assisted polymerization is an innovative technique that accelerates the polymerization process, making it both time-efficient and energy-saving. Microwave radiation promotes uniform heating, allowing for fast synthesis and improved control over the polymer’s structural uniformity. Hydrogels synthesized using microwave-assisted polymerization exhibit enhanced mechanical strength and are often used in heavy metal adsorption and environmental cleanup due to their durability [35].

Frontal polymerization offers a unique approach where a thermal wave, initiated at one end of the monomer mixture, propagates through the material, converting monomers into polymers. This method is known for creating highly homogenous hydrogels and is advantageous in synthesizing temperature-sensitive hydrogels used in drug delivery and responsive materials. This approach enables rapid synthesis while minimizing phase separation, which is ideal for generating consistent materials [36].

Photopolymerization is increasingly applied for hydrogels intended for biomedical applications, where spatial control is required. Using a light source to initiate polymerization provides high spatial precision, enabling hydrogel formation in specific areas. This method is particularly suited for in situ hydrogel formation, as it allows for precise placement in surgical or wound environments. Photo-crosslinked hydrogels are known for their mechanical robustness and tunable swelling properties, suitable for controlled drug release and tissue engineering applications [37].

Two-photon polymerization (TPP) is a laser-based technique that enables the fabrication of complex hydrogel structures at the microscale and nanoscale. This method relies on the nonlinear absorption of two photons to initiate polymerization, allowing for high-resolution patterning. Song et al. highlighted the potential of TPP in creating architecturally complex hydrogel scaffolds for tissue engineering applications, emphasizing its ability to produce intricate structures that mimic natural extracellular matrices [38]. The precision of TPP makes it a valuable tool in the field of hydrogel synthesis.

(ii) Cross-linking is essential for creating the three-dimensional network characteristic of hydrogels, and it can be achieved chemically or physically. Chemical cross-linking, often using agents like N,N′-methylenebisacrylamide, provides stable and durable hydrogels suitable for prolonged or repeated use, such as in drug delivery systems. Physical cross-linking, which relies on ionic or hydrogen bonding, is advantageous for applications requiring reversible properties and minimal chemical residues [39].
(iii) Grafting techniques involve attaching hydrogel networks onto other polymer backbones, enhancing their functionality, mechanical strength, and responsiveness. Graft copolymerization, a widely employed technique, combines different monomers to create hydrogels that respond to environmental stimuli, such as pH or temperature. For example, the grafting of polymethacrylamide onto sodium alginate forms pH-sensitive hydrogels with high swelling capacities, making them effective in controlled drug delivery applications [39].

These synthesis techniques enable the tailoring of hydrogel properties, such as porosity, mechanical strength, and responsiveness, to meet specific application requirements across various environmental and biomedical fields. Each method presents unique advantages, from high control over polymer structure to rapid synthesis and targeted functionality, further broadening the potential applications of hydrogels in advanced technology and sustainable solution.

### 2.2. Hydrogel Types

Hydrogels are three-dimensional (3D) networks capable of absorbing and retaining large amounts of water due to hydrophilic functional groups like -NH_2_, -OH, -COOH, -CONH, -CONH_2_, and -SO_3_H within their polymer structure [40,41,42]. These networks are composed of crosslinked polymer chains that are formed through physical or chemical cross-linking of natural or synthetic polymers [43,44]. Physical cross-linking involves temporary bonds such as hydrogen bonding or hydrophobic and electrostatic interactions, while chemical cross-linking creates permanent covalent bonds that are enhanced by ionic interactions [24]. The absorbed water is stored in the spaces between the crosslinked polymer chains, and hydrogels with a swelling capacity exceeding 100% are termed superabsorbent hydrogels (SHs) [45]. The properties of hydrogels, including swelling capacity, degradability, stiffness, and size, can be tailored by adjusting the hydrophilic–hydrophobic balance, polymer concentration, or reaction conditions [46].

**Figure 2:**
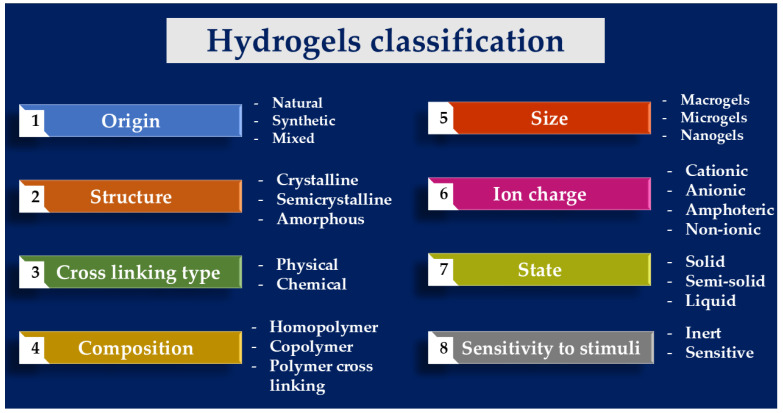
A comprehensive classification of hydrogels, highlighting various types based on their chemical composition, structure, and responsiveness to external stimuli.

Hydrogels are classified based on their material origin, polymer composition, configuration, cross-linking type, physical appearance, and ionic charge (Figure 2).

#### 2.2.1. Polymeric Composition

They can be derived from synthetic or natural sources, with natural hydrogels originating from biopolymers such as cellulose, starch, lignin, collagen, and alginate [41,42,43,47]. These natural hydrogels are biodegradable and biocompatible, making them suitable for biomedical applications, though they often face challenges related to mechanical strength and stability. In contrast, synthetic hydrogels, produced from petroleum-derived polymers like PVA, PEG, and PAAM, offer higher stability and mechanical strength [42,47]. They are customizable to meet specific application requirements, enhancing their suitability for uses such as drug delivery and implants [47].

Semisynthetic hydrogels combine attributes of both natural and synthetic hydrogels, and they are created by chemically modifying natural polymers or blending them with synthetic counterparts. Examples include GelMA (gelatine methacrylate) and AcHyA (hyaluronic acid acrylate). These materials retain the bioactivity of natural hydrogels while offering improved tunability, enabling precise control over their properties for diverse applications, including biomedical and environmental uses [40,43,44].

Hydrogels can also be categorized based on their chemical composition into homopolymers, copolymers, and interpenetrating polymer networks (IPNs) [48,49]. Homopolymer hydrogels are made from a single type of monomer, which serves as the primary structural unit. Copolymer hydrogels, or heteropolymers, consist of two or more different monomers arranged randomly or as repeating blocks, with at least one monomer being hydrophilic. IPN hydrogels are formed from interwoven chains of two or more polymers, which can be of natural or synthetic origin. In full IPNs, the polymers are independently crosslinked without forming shared chemical bonds [48,49]. In contrast, semi-IPNs consist of one crosslinked polymer intertwined with another polymer in a linear or branched form, which remains uncrosslinked and not chemically bonded to the first [48,49].

#### 2.2.2. Cross-Linking

Cross-linking is a fundamental aspect of hydrogel design, as it influences the network structure, mechanical properties, and responsiveness to stimuli. Hydrogels can be classified based on their type of cross-linking into physical and chemical categories. Physical hydrogels, also known as reversible hydrogels or pseudo-gels, are held together by temporary interchain interactions such as ionic, hydrogen, dipolar, or hydrophobic bonds. These bonds are sensitive to environmental conditions and can be disrupted by changes in factors like ionic strength, pH, or temperature. In contrast, chemical hydrogels are stabilized with permanent covalent bonds, which provide structural stability regardless of external environmental changes. These chemically crosslinked hydrogels are durable and decompose only through the complete breakdown of their macromolecules. Both types share features such as heterogeneous structures, uneven cross-linking, and non-uniform water distribution [50,51,52].

Research by Wu et al. highlighted the mechanical properties of covalently and physically crosslinked networks, emphasizing that the type of cross-linking significantly affects the hydrogel’s elasticity and recovery behavior [53]. Moreover, Li et al. explored bio-inspired polymer–nanoparticle bond dynamics, demonstrating that the interplay between different cross-linking types can be harnessed to tune hydrogel mechanics [54]. The choice of cross-linking method is therefore pivotal in designing hydrogels with desired properties.

#### 2.2.3. Network Electrical Charge

Their physical structure, charge, and particle size are also factors to consider when categorizing a hydrogel. Structurally, they are classified as amorphous (non-crystalline), crystalline, or semi-crystalline, with the latter combining both amorphous and crystalline phases. Based on the charge of their polymer chain side groups, hydrogels are divided into four types: anionic (negatively charged), cationic (positively charged), amphoteric (capable of forming both anions and cations), and nonionic (electrically neutral). Additionally, hydrogels are grouped by particle size into macrogels (>100 µm), microgels (0.1–100 µm), and nanogels (<0.1 µm) [55,56].

The electrical charge of the hydrogel network can also influence its swelling behavior, mechanical properties, and responsiveness to stimuli. Charged hydrogels, such as polyelectrolyte gels, exhibit unique behaviors in response to changes in ionic strength, pH, and temperature. The presence of charged groups can enhance the hydrogel’s ability to swell and respond to environmental stimuli, making them suitable for applications in drug delivery and biosensing [54,57]. For instance, Meng et al. reported on polypeptide-based supramolecular hydrogels mediated by Ca^2+^ ion cross-linking, highlighting how the network’s electrical charge can be tuned to achieve the desired mechanical properties and responsiveness [58]. Additionally, covalent cross-linking in charged hydrogels could improve their mechanical performance, indicating that the network’s electrical charge plays a significant role in determining the hydrogel’s overall behavior. The electrical charge of the hydrogel network is thus an important parameter that influences its functionality. Yang et al. demonstrated that covalent cross-linking in chitosan (C_56_H_103_N_9_O_39_) hydrogels resulted in a significant increase in Young’s modulus, indicating enhanced mechanical strength. The study highlighted that the electrical writing process induced covalent cross-linking, which formed a rigid chain network, thereby improving the hydrogel’s overall mechanical properties [59]. Additionally, Wu et al. explored the mechanical and adhesive properties of hydrogels that were crosslinked using both covalent and ionic methods. Their findings suggested that the combination of covalent cross-linking with ionic interactions led to hydrogels that exhibited superior mechanical strength and stability, further supporting the notion that the network’s electrical charge plays a significant role in determining the hydrogel’s behavior [60]. Moreover, Gu et al. investigated hybrid crosslinked polyacrylamide hydrogels, which incorporated ionic interactions into a covalent cross-linking network. Their results indicated that the mechanical properties of these hydrogels were significantly enhanced, demonstrating the importance of both covalent cross-linking and the presence of electrical charges in influencing hydrogel functionality [61].

In summary, classifying stimulus-responsive hydrogels based on polymeric composition, configuration, type of cross-linking, and network electrical charge provides a comprehensive understanding of their design and application potential. The interplay between these factors determines the mechanical properties, swelling behavior, and responsiveness of hydrogels to external stimuli. Continued research in this field is essential for the development of advanced hydrogels with tailored properties for specific applications.

In terms of biodegradability, hydrogels are classified as biodegradable or non-biodegradable. Biodegradable hydrogels break down into harmless low-molecular-weight products through enzymatic or microbial hydrolysis, making them environmentally friendly and biocompatible. Non-biodegradable hydrogels, however, retain their structure in biological and environmental settings [62,63].

Hydrogels can be categorized based on their aggregation state, biodegradability, and responsiveness to external stimuli. Regarding aggregation state, they are classified as solid, semi-solid, or liquid. Solid hydrogels have a highly crosslinked structure that is maintained by covalent bonds, making them swell in aqueous environments, which is ideal for medical and environmental applications [64]. Semi-solid hydrogels, with a looser network of weaker intermolecular interactions (e.g., van der Waals and hydrogen bonding), offer strong adhesive properties; they are suitable for prolonged drug delivery to soft tissues such as buccal, sublingual, ocular, or vaginal areas. Liquid hydrogels remain fluid at room temperature but exhibit elasticity and tissue-like functionality at specific temperatures [64].

Hydrogels are also divided into inert (conventional) and responsive (smart) types based on their sensitivity to external stimuli [64]. Conventional hydrogels are hydrophilic polymers, often nonionic, that swell significantly in water but lack adaptive functionality. Stimulus-responsive hydrogels, in contrast, adjust their properties, such as swelling, when exposed to changes in temperature, pH, ionic strength, electric or magnetic fields, mechanical stress, light, ultrasonic radiation, or specific substances (e.g., glucose, enzymes, or antigens). This adaptability makes them highly versatile for biomedical and environmental applications [64].

### 2.3. Functionalization for Enhanced Environmental Compatibility

For hydrogels, functionalization is particularly useful in enhancing their effectiveness for water purification and treatment processes. This can be achieved through two main approaches: (i) chemical modification of the polymeric hydrogel network, which alters its intrinsic structure to improve properties such as hydrophilicity, sorption capacity, or stability and (ii) immobilization of active compounds on the surface or within the porous hydrogel matrix, enabling the hydrogel to interact with specific contaminants and enhance its functional performance [65,66].

Chemical modifications of hydrogels are crucial for improving their stability, reusability, and environmental safety, particularly in applications related to wastewater treatment. Chemical modification involves altering the properties of hydrogels by introducing new compounds or functional groups through chemical reactions. This process allows for precise adjustments to the components of the three-dimensional network, forming permanent covalent bonds that modify the material’s physical and chemical characteristics. By employing targeted chemical modifications, hydrogels with enhanced surface hydrophilicity and improved sorption properties can be created [25].

For instance, the incorporation of chitosan into hydrogels has been shown to enhance their mechanical strength and adsorption capacity for pollutants due to the presence of functional groups such as -NH_2_ and -OH, which facilitate interactions with various contaminants [67,68]. Additionally, the use of composite hydrogels, such as those formed with alginate and polyvinyl alcohol (PVA) (C_2_H_4_O)_x_, has demonstrated improved viscoelastic properties and enhanced gel strength, making them more effective in pollutant removal [69,70].

Moreover, the development of self-healing hydrogels through dynamic covalent bonds allows for the recovery of mechanical properties after damage, thereby extending the lifespan of these materials in environmental applications [71,72]. For example, hydrogels that utilize the reversible unfolding and refolding of protein domains can achieve significant tunability in their mechanical properties, making them suitable for various environmental conditions [73]. Furthermore, the integration of cellulose nanofibrils into hydrogel matrices has been reported to enhance stability and strength, enabling these materials to withstand higher temperatures and harsh environmental conditions [74].

The immobilization of active molecules within hydrogel matrices is a widely used approach to endow hydrogels with specific functionalities [75]. This technique enhances processing efficiency and enables the neutralization of harmful compounds in aqueous solutions. Among the methods employed, the trapping technique is particularly popular due to its simplicity, speed, and cost-effectiveness. This method preserves the unique properties of the immobilized substances by suspending them within the free spaces of the crosslinked polymer network, which are filled with water, buffer solutions, or physiological fluids [75]. Through this process, hydrogels enriched with diverse additives become highly functional materials with broad application potential. A wide range of substances, including nanoparticles, metal oxides, antibodies, drugs, enzymes, organic compounds, fertilizers, plant protection agents, algae, microorganisms, and cells, has been successfully immobilized in hydrogel matrices, making them versatile for various biomedical, environmental, and agricultural uses [76,77,78].

For example, hydrogels with immobilized microorganisms have been effectively applied in various wastewater treatment contexts, including municipal systems (achieving reductions in BOD [79], COD, and protein content below recommended levels). In industrial wastewater, they support the biodegradation of p-cresol concentrations up to 200 mg/L, and in oily wastewater, they achieve a biodegradation rate of approximately 66.5% [79,80,81]. These hydrogels are also used to remove contaminants such as polycyclic aromatic hydrocarbons, nitrogen, heavy metals, phenolic compounds, and petroleum pollutants [82,83].

### 2.4. Stimuli-Responsiveness Optimization for Wastewater Treatment

**Figure 3:**
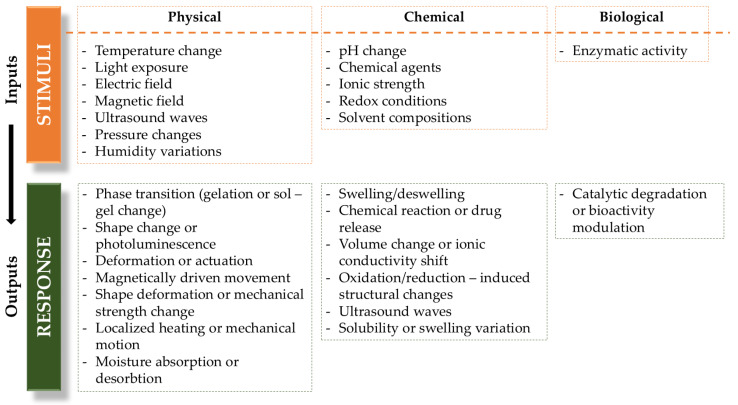
An overview of hydrogels’ reactions to different stimuli, illustrating their adaptability and functionality in response to environmental changes such as pH, temperature, and light.

Stimulus-responsive hydrogels can be classified into three primary categories based on the following types of stimuli: biological, chemical, and physical. Figure 3 demonstrates the ways in which these hydrogels react to various stimuli. Physical stimulus-responsive hydrogels react to signals like light and temperature; chemical stimulus-responsive hydrogels respond to factors such as ions and pH levels, while biological stimulus-responsive hydrogels are sensitive to biomolecules, including enzymes and sugars.

Physical signals, such as temperature and light, offer significant advantages for monitoring due to their non-invasive and controllable nature, without introducing complex substances that could interfere with signal output. Hydrogels responsive to physical stimuli have found extensive applications. For example, Hou et al. developed self-healing hydrogels with reversible thermal responsiveness, leveraging a cross-linking-induced thermal response mechanism [84]. These hydrogels had a lower critical solution temperature set at approximately 37 °C, making them promising carriers for controlled drug release. Similarly, Park et al. synthesized Py–CN–MBE/PAA photoresponsive polymer gels with strong fluorescence properties. Upon UV irradiation, these gels transitioned from a gel to a sol state while emitting a faint blue fluorescence, demonstrating potential for applications in drug delivery, photography, optical storage, biomedical imaging, and biosensors [85].

Chemical stimulus-responsive hydrogels, containing functional groups such as amino, pyridine, carboxylic acid amide, and hydrazone bonds, react to changes in ion concentration within a solution. For instance, Yue et al. developed core–shell hydrogel nanoparticles with pH-responsive properties, enabling the sensitive detection of cancer cells by distinguishing the pH levels between normal and tumor tissues [86]. Similarly, Yang et al. designed a supramolecular hydrogel (MQ-G) with ion-responsive capabilities, effectively identifying iron ions and hydrogen phosphate in water. These hydrogels hold potential not only for disease detection but also for applications in food safety monitoring [87].

Biochemical stimulus-responsive hydrogels, as a novel intelligent material, have garnered significant attention due to their exceptional properties. Huang et al. developed an ATPase-responsive hydrogel self-assembled from sodium polystyrene sulfonate and tetrastyrene derivatives [88,89]. The presence of ATP disrupts molecular interactions, inducing a gel-sol transition, while ATPase decomposes ATP, triggering a sol–gel transformation. This responsiveness makes the hydrogel suitable for imaging biological oxidation processes in cytoplasm. Additionally, Heilshorn et al. created an intelligent hydrogel by assembling DNA molecules, which released antimicrobial peptides upon exposure to pathogenic *Staphylococcus aureus*. This targeted response enables rapid antibacterial action at infection sites. Such biochemical stimulus-responsive hydrogels hold great promise for biosensing applications in food safety [90].

Stimulus-responsive hydrogels exhibit unique adaptability due to their chemical composition, allowing them to respond dynamically to environmental changes. The presence of specific functional groups, such as carboxyl, amine, sulfonate, and hydroxyl, plays a crucial role in determining their swelling behavior, adsorption capacity, and overall performance. Understanding these chemical groups and their interactions enhances the development of advanced hydrogels for applications in wastewater treatment, biomedical engineering, and smart materials. The following discussion highlights the key functional groups responsible for hydrogel responsiveness and their practical implications (Table 1).

Tailoring the properties of hydrogels, such as pore size and surface area, is essential for optimizing their performance in wastewater treatment applications. The design of hydrogels with specific morphologies, such as bilayer structures, can facilitate localized bending and deformation in response to external stimuli, enhancing their adaptability to varying environmental conditions [101]. For instance, hydrogels engineered to respond to changes in pH or temperature can exhibit significant swelling or deswelling, which is beneficial for the selective adsorption and release of pollutants [102,103].

Moreover, the incorporation of metal ions or nanoparticles into hydrogel networks can dynamically regulate their mechanical properties and responsiveness, allowing for enhanced pollutant capture and removal efficiency [104,105]. For example, the use of calcium alginate and bentonite in composite hydrogels has been shown to improve adsorption performance for heavy metals, demonstrating the importance of tailoring hydrogel composition for specific environmental needs [105]. Additionally, the development of mechano-responsive hydrogels that can detect and respond to mechanical stimuli offers new avenues for enhancing the effectiveness of wastewater treatment processes [106,107].

The functionalization and optimization of hydrogel properties are critical for enhancing their environmental compatibility and responsiveness in wastewater treatment applications. By employing various chemical modifications and tailoring hydrogel structures, researchers can develop more effective materials for addressing the challenges posed by pollutants in wastewater.

## 3. Mechanisms of Pollutant Removal Using Stimulus-Responsive Hydrogels

### 3.1. Adsorption Mechanisms and Surface Interactions

**Figure 4:**
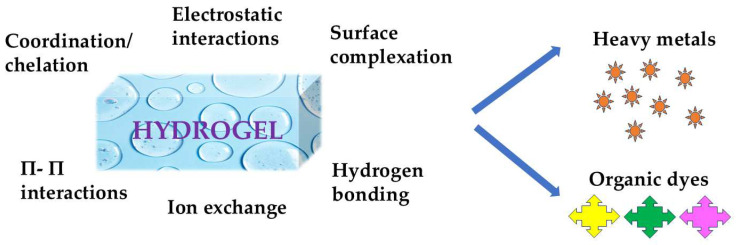
A schematic illustration for the adsorption mechanism.

Stimulus-responsive hydrogels exhibit various adsorption mechanisms that facilitate the removal of pollutants from aqueous environments (Figure 4). Key interactions include hydrophobic interactions, ion exchange, and electrostatic interactions. Hydrophobic interactions play a significant role in the adsorption of organic pollutants, as non-polar molecules tend to associate with the hydrophobic regions of the hydrogel matrix, enhancing their removal efficiency [108].

For instance, hydrogels composed of poly(N-isopropylacrylamide) (PNIPAM) have been shown to effectively capture hydrophobic contaminants due to their ability to undergo phase transitions in response to temperature changes, which alters the hydrophobicity of the hydrogel surface [108].

Ion exchange mechanisms are also critical, particularly for the removal of heavy metal ions. Hydrogels containing functional groups such as carboxylates or sulfonates can interact with metal ions through ion exchange processes, effectively sequestering these pollutants from water [109]. Electrostatic interactions further enhance adsorption capabilities, especially for charged pollutants. For example, hydrogels with cationic or anionic properties can attract oppositely charged contaminants, thereby increasing their adsorption capacity [110]. The combination of these interactions allows for a versatile approach to pollutant removal, making stimulus-responsive hydrogels effective materials for environmental remediation.

### 3.2. Stimulus-Induced Changes to Enhance Removal Efficiency

The responsiveness of hydrogels to external stimuli, such as temperature, pH, and light, significantly enhances their pollutant capture efficiency. When exposed to specific stimuli, hydrogels can undergo swelling or deswelling, which alters their pore size and surface area, thereby facilitating the uptake of pollutants. For instance, temperature-responsive hydrogels, such as those based on PNIPAM, exhibit a sharp volume phase transition at their lower critical solution temperature (LCST). This transition allows the hydrogel to swell and increase its surface area, leading to improved adsorption of organic pollutants [108].

Moreover, the morphological changes induced by external stimuli can create dynamic environments that enhance pollutant capture. For example, hydrogels that undergo shape changes in response to pH variations can expose additional adsorption sites, thereby increasing their capacity for pollutant removal [111]. Additionally, the incorporation of nanoparticles, such as graphene oxide or carbon nanotubes, into hydrogel matrices can further enhance responsiveness and adsorption efficiency by providing additional active sites for pollutant interaction [109,112]. These stimulus-induced changes not only improve the efficiency of pollutant capture but also enable the development of smart hydrogels that can adapt to varying environmental conditions.

### 3.3. Release Mechanisms for Reusability

The ability to release captured pollutants is crucial for the reusability of stimulus-responsive hydrogels. External stimuli can trigger the release of adsorbed contaminants, allowing the hydrogels to be reused for subsequent pollutant removal cycles. For instance, hydrogels that respond to temperature changes can release captured pollutants upon cooling, as the hydrogel contracts and reduces its affinity for the adsorbed molecules [108]. Similarly, pH-responsive hydrogels can release pollutants when the pH of the surrounding environment is altered, causing a change in the hydrogel’s ionic interactions and swelling behavior [111].

Light-triggered release mechanisms have also been explored, where hydrogels containing photoresponsive components can degrade or alter their structure upon exposure to specific wavelengths of light, facilitating the release of captured pollutants [110]. This feature is particularly advantageous for applications requiring precise control over pollutant release, such as in situ remediation efforts. By employing these release mechanisms, stimulus-responsive hydrogels can be effectively reused, reducing waste and enhancing their sustainability in environmental applications.

The mechanisms of pollutant removal using stimulus-responsive hydrogels involve a combination of adsorption interactions, stimulus-induced morphological changes, and effective release strategies. These features make them promising candidates for addressing environmental pollution challenges.

## 4. Applications of Stimulus-Responsive Hydrogels in Wastewater Treatment: Case Studies

Due to their responsive nature, stimulus-responsive hydrogels have gained significant attention for wastewater treatment by offering targeted pollutant removal, improved filtration efficiency, and enhanced water recovery processes. Their ability to adjust their physicochemical properties allows advanced wastewater treatment systems to precisely control pollutant capture and release, making them highly effective in diverse environmental conditions.

Table 2 illustrates the applications of stimulus-responsive hydrogels in wastewater treatment, along with relevant case studies from the provided literature.

### 4.1. Heavy Metal Ion Removal

To be widely effective in removing heavy metals from polluted wastewater, an ideal hydrogel should possess the following properties [25]:

- Cost-effectiveness: affordable to produce and implement on a large scale;
- High adsorption capacity: capable of efficiently capturing heavy metals from wastewater;
- Fast adsorption rate: enhanced by optimal porosity and particle size for rapid heavy metal uptake;
- Biodegradability: environmentally friendly and capable of decomposing naturally after use;
- Ease of modification: allows for chemical and structural adjustments to enhance performance;
- Low residual monomer content: contains minimal amounts of unreacted monomers, ensuring safer application;
- Stability and durability: maintains functionality and structural integrity during swelling and long-term storage;
- Non-toxic, colorless, and odorless: safe and pleasant to handle without undesirable chemical characteristics;
- pH neutrality: remains chemically stable without affecting the pH of the aqueous environment after swelling;
- Deswelling and rehydration ability: capable of releasing stored water and reabsorbing it when needed.

Stimulus-responsive hydrogels have shown significant promise in the removal of heavy metal ions such as lead, cadmium, and mercury from wastewater. These hydrogels often incorporate functional groups that facilitate ion exchange and complexation, enhancing their capacity to sequester toxic metals.

For instance, a study demonstrated that hydrogels modified with carboxyl and amine groups effectively removed lead ions from aqueous solutions through electrostatic interactions and ion exchange mechanisms [123]. The incorporation of metal-binding ligands into the hydrogel matrix can further enhance their selectivity and efficiency for specific metal ions, making these materials highly effective for environmental remediation.

Moreover, the responsiveness of these hydrogels to external stimuli, such as pH or temperature, allows for dynamic control over their adsorption properties. For example, pH-sensitive hydrogels can swell or shrink in response to changes in the surrounding environment, thereby exposing more active sites for metal ion binding during the adsorption phase [124]. This tunability not only increases the efficiency of heavy metal removal but also enables the hydrogels to be regenerated and reused, thus reducing waste and operational costs associated with wastewater treatment processes [125].

**Figure 5:**
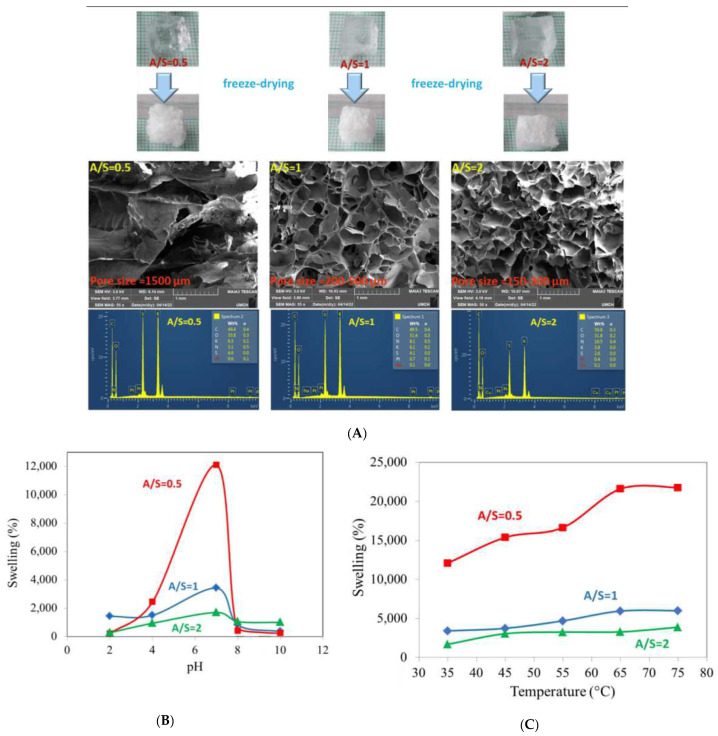
(**A**) The HPMC-g-poly(AM-co-SPA) hydrogels were examined both before and after freeze-drying, with SEM images revealing their porous morphology and EDX analysis providing elemental composition details; (**B**) the pH-responsive behavior of the HPMC-g-poly(AM-co-SPA) hydrogels; (**C**) temperature-responsive behavior of HPMC-g-poly(AM-co-SPA) hydrogels [126].

In a recent study, the authors developed highly swelling, pH, and temperature stimuli-responsive hydrogels for the efficient removal of inorganic pollutants from water (Figure 3) [126]. Hydroxypropyl methyl cellulose (HPMC) was grafted with acrylamide (AM) and 3-sulfopropyl acrylate (SPA) via radical polymerization and crosslinked with a di-vinyl comonomer (Figure 5). The hydrogel with the highest SPA content (AM/SPA ratio = 0.5) exhibited superior performance, including a 12,100% swelling ratio, rapid kinetics, and strong temperature and pH responsiveness, though with lower mechanical strength. Hydrogels with AM/SPA ratios of one and two showed higher mechanical moduli but reduced responsiveness. Cr(VI) adsorption tests demonstrated 90–96% removal efficiency, with hydrogels at AM/SPA ratios of 0.5 and 1 showing promise for pH-regenerable, repeated use.

### 4.2. Organic Pollutant and Dye Removal

The discharge of dye-contaminated wastewater from industries like textiles, paper, plastics, and leather poses a serious threat to aquatic ecosystems and human health. With over 10,000 dyes produced annually, this leads to the release of tons of dye pollutants into the environment, and the consequences are significant. The application of stimulus-responsive hydrogels for the removal of organic pollutants, including dyes, pesticides, and pharmaceuticals, has garnered considerable attention in recent years. These hydrogels can effectively adsorb a wide range of organic contaminants due to their high surface area and tunable porosity [127]. Van Tran et al. reviewed the use of hydrogels as versatile adsorbents for eliminating contaminants from water and wastewater [128]. Hydrogels primarily remove aqueous pollutants through mechanisms such as electrostatic interactions, hydrogen bonding, and hydrophobic interactions [47].

The complex chemical structures of organic compounds, often containing aromatic and azo groups, make them highly resistant to degradation by water and light. Compounds such as naphthalene and benzidine derivatives further exacerbate their environmental and health impacts [129]. Many of these dyes are toxic, carcinogenic, and mutagenic to aquatic organisms [130].

To address dye pollution, various removal techniques have been employed, including flocculation–precipitation [131], photocatalytic and sonocatalytic oxidation [132,133,134], Fenton oxidation [135], electrochemical degradation [136,137], electrocatalytic oxidation [136], and adsorption using materials like clay [138] or biomass-derived activated carbon [139]. Among these, adsorption stands out as the most efficient and cost-effective method for removing dyes from wastewater. Its advantages include simplicity, economic feasibility, and effectiveness in eliminating a wide range of dye pollutants [128].

This study presents a novel wood-based photocatalytic system for efficient wastewater treatment, hydrogen production, and freshwater generation. By combining photoactive CdS with 2D MoSe_2_ nanosheets in delignified wood and embedding a PVA–chitosan hydrogel for stability and hydrophilicity, the system accelerates water evaporation while reducing volatile organic compound contamination. It achieves a hydrogen evolution rate of 9.7 mmol g^−1^ h^−1^ and a solar evaporation rate of 1.92 kg m^−2^ h^−1^, with 90.7% energy conversion efficiency under one-sun illumination. The integrated photocatalytic and photothermal functionalities provide a sustainable, high-performance solution for water purification and renewable energy [140]. 

For instance, hydrogels synthesized from poly(2-hydroxyethyl methacrylate) (pHEMA) and N-isopropylacrylamide (NIPAM) have been shown to exhibit excellent adsorption capacities for methylene blue and other organic dyes, with the adsorption process being influenced by temperature and pH [123].

**Figure 6:**
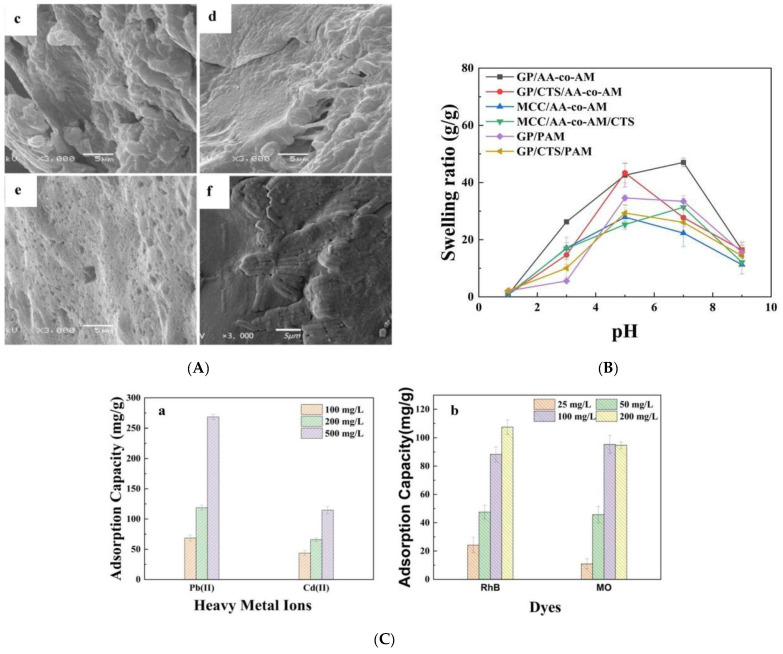
(**A**) The SEM images represent the morphological structures of various hydrogels synthesized through radical polymerization: (**c**) GP/AA-co-AM, (**d**) GP/GTS/AA-co-AM, (**e**) GP/PAM, and (**f**) MCC/AA-co-AM; (**B**) swelling ratios of hydrogels at different pHs; (**C**) the competitive adsorption behavior of GP/CTS/AA-co-AM was evaluated for (**a**) Pb(II), Cd(II), (**b**) RhB, and MO in mixed solutions [141].

Yin et al. developed hydrogel adsorbents for water pollution remediation using Guipi residue (GP), a cellulose material from Chinese herbal medicine, and chitosan (CTS) via radical polymerization with acrylamide (AM) and acrylic acid (AA) [141] (Figure 6). The GP/CTS/AA-co-AM hydrogel exhibited enhanced mechanical properties and effectively removed pollutants such as Pb^2+^, Cd^2+^, Rhodamine B (RhB), and methyl orange (MO). At pH 4.0, with a contact time of 120 min, the maximum adsorption capacities were 314.6 mg/g for Pb^2+^, 289.1 mg/g for Cd^2+^, 106.4 mg/g for RhB, and 94.8 mg/g for MO, maintaining stability across pH levels. The hydrogel showed superior selectivity for Pb^2+^ and followed pseudo-second-order kinetics and the Langmuir isotherm model. After five adsorption–desorption cycles, the hydrogel retained over 70% capacity for metals and 80% for dyes, demonstrating its efficiency and reusability for water treatment applications.

The incorporation of photocatalytic materials into hydrogel structures has also been explored to enhance the degradation of organic pollutants. Photocatalytic hydrogels can harness light energy to initiate chemical reactions that break down organic contaminants, thereby improving overall removal efficiency [124]. This dual functionality of adsorption and photocatalysis makes these hydrogels particularly attractive for treating complex wastewater streams that contain a mixture of pollutants.

**Figure 7:**
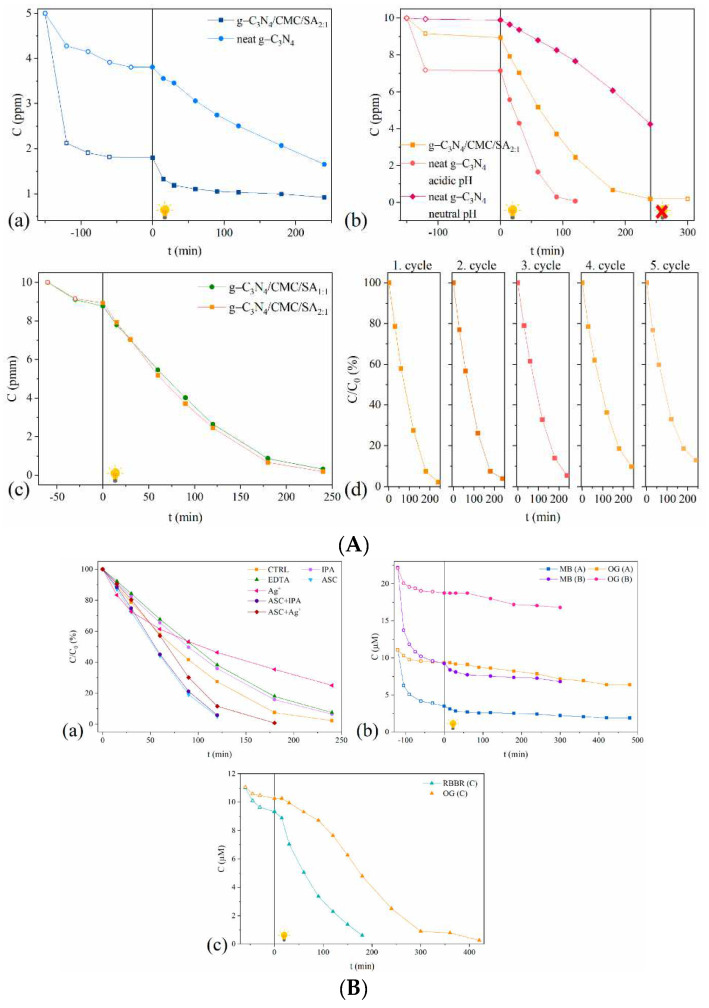
(**A**) Photodegradation of (**a**) MB dye and (**b**) OG dye using pristine g-C3N4 and g-C_3_N_4_/CMC/SA_2:1_ beads; (**c**) OG dye degradation using g-C_3_N_4_/CMC/SA_1:1_ and g-C_3_N_4_/CMC/SA_2:1_ beads; and (**d**) OG dye degradation using g-C_3_N_4_/CMC/SA_2:1_ beads over 5 consecutive cycles [Experimental conditions: initial concentration of OG = 10 ppm, MB = 5 ppm, pH = 3.8, temperature = 25 °C, under simulated solar light]; (**B**) (**a**) Photodegradation performance of g-C3N4/CMC/SA2:1 beads was evaluated under the influence of various scavengers and reactive agents, including IPA, EDTA, ASC, Ag^+^, IPA/ASC, ASC/Ag^+^, and EDTA/ASC. The experimental setup involved OG dye with an initial concentration of 10 ppm at pH 3.8, a temperature of 25 °C, and simulated solar light irradiation. (**b**) Adsorption and photodegradation efficiency of MB and OG dyes were assessed in binary-dye solutions, considering their different charge properties and interaction potentials with the composite beads. (**c**) Similarly, RBBR and OG dyes were tested in binary-dye solutions to explore the composite’s ability to manage competitive adsorption and photodegradation under similar experimental conditions [142].

A recent study focused on the eco-friendly synthesis of g-C3N4/CMC/SA composite hydrogels using a simple two-step cross-linking process [142]. The encapsulation of g-C3N4 nanoparticles into crosslinked carboxymethyl cellulose (CMC) and alginate (SA) copolymers resulted in composite hydrogels with enhanced porosity, thermal stability, photostability, and strong mechanical integrity (Figure 7). The coherent structure and strong adhesion of g-C3N4 within the CMC/SA matrix ensured long-term stability and recyclability. The composite hydrogels demonstrated selective adsorption and photocatalytic degradation of structurally different dyes with opposite charges, including cationic methylene blue (MB) and anionic dyes like Orange G (OG) and Remazol Brilliant Blue R (RBBR), in both single- and binary-dye systems. The adsorption and photocatalytic performance were strongly influenced by dye structure and charge interactions. OG and RBBR mainly interacted with g-C3N4, while MB was adsorbed by the negatively charged CMC/SA copolymers. The hydrogels showed high photocatalytic efficiency toward OG and a significant adsorption of MB. Scavenger studies confirmed that photo-induced electrons were the primary reactive species responsible for OG degradation under solar light. In binary-dye systems, MB reduced OG degradation via competitive adsorption, while RBBR exerted a stronger inhibitory effect on OG in anionic–anionic dye combinations. Leaching tests demonstrated the hydrogel’s long-term stability and minimal catalyst loss.

In recent years, bio-based hydrogels have emerged as promising adsorbents for dye removal due to their interconnected porous structure, high specific surface area, and enhanced adsorption performance. Functional groups such as carboxyl, hydroxyl, amine, and thiol in bio-based hydrogels have been tailored to improve dye adsorption. Examples of such hydrogels include modified forms of cellulose, chitosan, starch, sodium alginate (SA), and natural gums. These materials offer an environmentally friendly and efficient solution for addressing the persistent problem of dye contamination in water.

Viscusi et al. introduced an innovative, eco-friendly, and sustainable approach to efficiently remove methylene blue from water using halloysite as a nanofiller. Their system was created by blending sodium alginate with soybean extract; the product was reinforced with hemp hurd (an agricultural byproduct) and halloysite nanotubes. The composite beads were fabricated through the ionotropic gelation method, employing calcium chloride as a cross-linking agent. The study showed that incorporating 35% by weight of halloysite nanotubes significantly improved the adsorption capacity of methylene blue, increasing it from 32 mg/g to 49 mg/g due to the enhanced inorganic filler content [143].

Separately, Shahinpour et al. developed and characterized a high-performance magnetic bio-based hydrogel composite (CKAlFe), made from κ-carrageenan, kaolinite, Al_2_O_3_, and Fe_3_O_4_, to adsorb dyes such as Alizarin Red S and Congo Red from water. Alumina-coated iron oxide core–shell nanoparticles served as the magnetic component, enhancing chemical stability and providing additional adsorption sites. The porous polysaccharide and clay coating facilitated targeted adsorption in the diffusion medium and enabled composite separation via an external magnetic field. Morphological analysis confirmed the successful synthesis of the non-rigid, microporous CKAlFe composite, which featured κ-carrageenan as the amorphous phase and kaolinite clay, Al_2_O_3_, and Fe_3_O_4_ as the crystalline phases. The composite demonstrated impressive adsorption capacities, achieving maximum removal efficiencies of 26.9 mg/g for Congo Red and 33.5 mg/g for Alizarin Red S in binary solutions [144].

Baigorria et al. developed hybrid chitosan/clay nanocomposite hydrogels as potential biosorbents for paraquat in water. These materials were synthesized through straightforward procedures, producing nanocomposite beads with semi-spherical shapes and micrometer-scale dimensions. Physicochemical and morphological analyses revealed that the inclusion of nanoreinforcements interacted with the chitosan biopolymer matrix, significantly modifying its structure and properties. The beads had a highly porous structure with interconnected pores. Sorption studies of paraquat in water were conducted on nanocomposite beads modified with dodecylamine (DDA), dellite LVF (LVF), and bentonite (Bent), achieving maximum adsorption capacities of 0.98, 0.94, and 0.99 mg/g, respectively. The findings emphasized that incorporating nanoclays into the chitosan matrix is essential for effective paraquat biosorption [145].

Ngwabebhoh et al. studied the performance of a semi-interpenetrating network (IPN) superabsorbent chitosan–starch hydrogel for removing Direct Red 80 dye from water. The adsorption equilibrium conformed to the Freundlich isotherm model, demonstrating a maximum adsorption capacity of 312.77 mg/g, primarily driven by chemisorption mechanisms. This study highlights the effectiveness of combining biopolymeric matrices for dye removal [146].

In another study, Kang et al. utilized hydrogels composed of montmorillonite nanosheets (MMTs) and chitosan (CS) to adsorb methylene blue from aqueous solutions, achieving a maximum adsorption capacity of 530 mg/g. The adsorption process involved hydrogen bonding and cation exchange, with the structural configuration of the hydrogels playing a key role. Hydrogels with lower concentrations of MMT exhibited a less dense, stratified structure, enhancing their ability to adsorb methylene blue S [147].

Zhang et al. investigated the adsorption of methylene blue using a PAA/CS/Fe_3_O_4_ hydrogel fabricated through free radical polymerization, followed by treatment with Fe^2+^/Fe^3+^ and NaOH solutions. The PAA/CS/Fe_3_O_4_ hydrogel demonstrated a significantly higher adsorption capacity of approximately 1603 mg/g compared to hydrogels without Fe_3_O_4_. This remarkable performance was attributed to a high swelling ratio, strong electrostatic interactions, and the incorporation of magnetic Fe_3_O_4_ nanoparticles, which enhanced the material’s adsorption efficiency [148].

### 4.3. Oil and Grease Separation in Industrial Wastewater

The rapid growth of industrialization has led to the generation of vast quantities of oily wastewater, posing serious threats to the environment and human health. Hydrogels have also been effectively utilized in the separation of oil and grease from industrial wastewater, particularly in scenarios involving oil spills and oily effluents from food processing industries. Recent advancements in hydrogel technology have led to the development of zwitterionic hydrogel coatings that exhibit low adhesion to oils, facilitating the efficient separation of oil–water mixtures [149]. These hydrogels can be integrated into membrane systems, where they function as selective barriers that allow water to pass through while capturing oil and grease.

Zhao et al. developed a thermo-responsive cellulose-based hydrogel that alternates between superhydrophilic and hydrophobic states at different temperatures. This hydrogel effectively separated oil from water-rich or oil-rich mixtures, with high reusability and absorption capacities exceeding 10 times its weight. The material’s biodegradable nature adds to its environmental appeal [24]. A temperature-sensitive nanofibrous membrane grafted with poly(N-isopropylacrylamide) (PNIPAAm) exhibited switchable hydrophilic and hydrophobic properties, enabling controlled oil/water separation. The material achieved superior separation performance, especially useful for wastewater treatment applications [150].

A hydrogel film fabricated using 2-(dimethylamino)ethyl methacrylate and methacrylic acid demonstrated dual thermo- and pH-responsiveness. This hydrogel separated oil/water mixtures with a high separation efficiency of 98.35%, even after 15 cycles [151].

Hydrogels integrating DNA cross-linking units demonstrated switchable properties controlled by light and pH stimuli. These materials provided excellent selectivity and reusability in separating oil/water mixtures while enabling multifunctional applications [152].

A UV-responsive hydrogel, integrating azobenzene-modified cellulose, exhibited reversible transitions between hydrophilic and hydrophobic states upon UV exposure. This characteristic allowed for controlled oil/water separation, demonstrating high efficiency and reusability in multiple cycles. The material’s eco-friendly nature and ease of fabrication make it a potential game-changer for smart wastewater purification systems [153]. In another study, a nonwoven fabric coated with cellulose nanocrystals and triblock polymer brushes demonstrated reversible wettability under pH and UV-light stimuli. The material efficiently separated oil/water mixtures and degraded organic pollutants, showcasing multifunctional wastewater treatment capabilities [154].

Magnetic hydrogels fabricated using Fe_3_O_4_ nanoparticles embedded within a polymer matrix achieved efficient oil/water separation, leveraging external magnetic fields for easy recovery. These hydrogels maintained high separation efficiency even after prolonged use, showcasing durability and cost-effectiveness for large-scale applications [3].

The ability of hydrogels to undergo swelling in the presence of water while repelling oil makes them ideal candidates for applications in oily wastewater treatment. For example, a study highlighted the use of hydrogel-coated membranes that demonstrated high separation efficiency without the need for external pressure, allowing for easy cleaning and reuse after each separation cycle [149]. This characteristic not only enhances the sustainability of the treatment process but also reduces operational costs associated with traditional oil separation methods.

### 4.4. Pathogen and Microbial Control

The potential of stimulus-responsive hydrogels in pathogen and microbial control is an emerging area of research with significant implications for wastewater treatment. These hydrogels can be engineered to release antimicrobial agents in response to specific stimuli, effectively neutralizing bacteria, viruses, and other pathogens present in wastewater. For instance, hydrogels that release silver nanoparticles upon exposure to certain environmental triggers have been shown to exhibit potent antibacterial activity, thereby reducing microbial contamination in treated water.

Table 3 summarizes various applications of stimulus-responsive hydrogels in controlling different types of pathogens, highlighting their mechanisms of action and potential uses in various fields.

Additionally, the incorporation of bioactive compounds into hydrogel matrices can enhance their efficacy in pathogen removal. Recent studies have demonstrated that hydrogels loaded with probiotics or natural antimicrobial agents can effectively capture and neutralize harmful microorganisms in wastewater, promoting a safer and more sustainable treatment process [128]. The ability to tailor the release of these agents based on environmental conditions further enhances the effectiveness of hydrogels in controlling microbial populations in wastewater systems.

The applications of stimulus-responsive hydrogels in wastewater treatment are diverse and promising. Their ability to remove heavy metals, organic pollutants, oils, and pathogens highlights their potential as effective materials for addressing various environmental challenges. Continued research and development in this field will likely lead to more innovative solutions for sustainable wastewater management.

## 5. Limitations, Future Directions, and Prospects for Stimulus-Responsive Hydrogels in Environmental Science

Responsive hydrogels have emerged as a significant innovation in the field of wastewater treatment, owing to their unique properties that allow them to respond dynamically to environmental stimuli such as temperature, pH, and ionic strength. These materials are characterized by their ability to swell or shrink in response to changes in their surroundings, which can be harnessed for the efficient removal of pollutants from wastewater. The advantages of responsive hydrogels in water treatment are manifold, including their high adsorption capacity, tunable properties, and potential for regeneration and reuse. However, there are also limitations associated with their application, such as the complexity of synthesis, potential leaching of toxic components, and challenges related to scalability and economic feasibility. One of the primary advantages of responsive hydrogels is their high adsorption capacity for various pollutants, including heavy metals and organic dyes. For instance, studies have demonstrated that hydrogels can effectively absorb and accumulate contaminants from aqueous solutions, making them suitable for wastewater treatment applications [1,161]. The incorporation of nanomaterials into hydrogel matrices has further enhanced their adsorption capabilities. For example, nanocomposite hydrogels have been shown to exhibit improved mechanical properties and higher adsorption rates due to the synergistic effects of the nanomaterials [1,162]. This is particularly beneficial for treating industrial wastewater, which often contains a complex mixture of pollutants that require efficient removal strategies. Moreover, the tunability of responsive hydrogels allows for the optimization of their properties to suit specific treatment needs. By modifying the chemical composition and cross-linking density of the hydrogels, researchers can tailor their swelling behavior and responsiveness to environmental stimuli [163,164]. This adaptability is crucial in addressing the varying conditions encountered in wastewater treatment processes. For instance, pH-responsive hydrogels can selectively adsorb cationic or anionic dyes depending on the pH of the wastewater, thus enhancing the specificity of pollutant removal [165]. Additionally, the incorporation of stimuli-responsive elements enables the development of smart hydrogels that can release adsorbed pollutants upon exposure to specific triggers, facilitating the regeneration of the hydrogel for repeated use [166]. The self-healing properties of certain hydrogels also contribute to their advantages in water treatment applications. These materials can recover from physical damage, maintaining their structural integrity and functionality over time [1]. This characteristic is particularly advantageous in environments where mechanical stress is prevalent, as it extends the lifespan of the hydrogel and reduces the need for frequent replacements. Furthermore, the ability of hydrogels to undergo rapid swelling and deswelling cycles enhances their efficiency in capturing and releasing contaminants, thereby improving the overall treatment process [167]. Despite these advantages, there are notable limitations associated with the use of responsive hydrogels in wastewater treatment. One significant challenge is the complexity involved in synthesizing these materials. The production of hydrogels with specific responsive properties often requires intricate chemical processes that can be time-consuming and costly [163,168]. Additionally, the scalability of hydrogel production remains a concern, as many synthesis methods are not easily adaptable for large-scale applications. This limitation can hinder the widespread adoption of responsive hydrogels in industrial settings, where cost-effectiveness and efficiency are paramount. Another critical limitation is the potential leaching of toxic components from the hydrogel matrix into the treated water. Some hydrogels are synthesized using materials that may not be entirely biocompatible or environmentally friendly, raising concerns about the safety of using these materials in water treatment applications [169]. It is essential to ensure that the hydrogels do not release harmful substances during their operation, as this could negate the benefits of using them for pollution control. Moreover, while responsive hydrogels demonstrate impressive adsorption capacities, their performance can be influenced by various factors such as temperature, ionic strength, and the presence of competing ions in the wastewater [161,164]. These variables can affect the swelling behavior and, consequently, the adsorption efficiency of the hydrogels. Therefore, understanding the interactions between the hydrogel and the wastewater matrix is crucial for optimizing their performance in real-world applications. In conclusion, responsive hydrogels present a promising solution for wastewater treatment, offering significant advantages such as high adsorption capacity, tunable properties, and self-healing capabilities. However, challenges related to synthesis complexity, potential toxicity, and performance variability must be addressed to fully realize their potential in practical applications. Future research should focus on developing more efficient synthesis methods, exploring environmentally friendly materials, and conducting comprehensive studies on the long-term performance and safety of these hydrogels in wastewater treatment scenarios.

Integrating stimulus-responsive hydrogels with nanomaterials and bio-based polymers is paving the way for innovative solutions in environmental science. Recent advancements have demonstrated that hybrid hydrogels, which combine traditional polymer matrices with nanomaterials such as graphene oxide or titanium dioxide, exhibit enhanced mechanical properties and improved pollutant adsorption capabilities [170,171]. These nanocomposite hydrogels not only retain the desirable characteristics of hydrogels, such as high water retention and flexibility, but also leverage the unique properties of nanomaterials, including increased surface area and catalytic activity, to enhance pollutant degradation [170].

Additionally, the incorporation of bio-based polymers into hydrogel formulations can contribute to sustainability goals by utilizing renewable resources and reducing reliance on synthetic materials. For example, hydrogels derived from natural polysaccharides like alginate and chitosan have shown promise in wastewater treatment applications due to their biocompatibility and biodegradability [172]. The development of hybrid systems that combine bio-based polymers with synthetic components can further optimize the performance of hydrogels in environmental applications, leading to more effective and sustainable solutions for pollutant removal.

Stimulus-responsive hydrogels can play a crucial role in promoting sustainability and circular economy principles. By enabling the efficient removal of pollutants from wastewater, these materials contribute to waste minimization and resource recovery. For instance, hydrogels designed for the selective capture of heavy metals can facilitate the recycling of valuable resources from industrial effluents, thereby reducing environmental contamination and promoting resource efficiency [173].

Moreover, the reusability of hydrogels, facilitated by their responsiveness to external stimuli, aligns with circular economy principles by allowing for multiple cycles of pollutant capture and release without significant degradation of the material [172]. This characteristic not only reduces waste but also lowers operational costs associated with wastewater treatment processes. As the demand for sustainable solutions in environmental management continues to grow, the development of hydrogels that can effectively integrate into circular economy frameworks will be essential.

As the application of stimulus-responsive hydrogels in environmental science expands, future regulatory considerations will be critical to ensure their safety and efficacy. Regulatory bodies will need to establish guidelines for the use of these materials in wastewater treatment, particularly concerning their biodegradability, potential ecological impacts, and the safety of any embedded agents used for pollutant capture [174].

The standardization of testing methods for evaluating the performance of hydrogels in real-world applications will also be necessary to facilitate the comparison of different hydrogel formulations and their effectiveness in pollutant removal [175]. Furthermore, addressing public concerns regarding the environmental impact of synthetic materials will be essential for the widespread adoption of hydrogels in environmental applications.

Despite their potential, several challenges and limitations must be addressed to enhance the effectiveness of stimulus-responsive hydrogels in environmental applications.

The stability and biodegradability of hydrogels are critical factors that influence their environmental impact. While many hydrogels are designed to degrade under specific conditions, the rate and byproducts of degradation can vary significantly depending on the hydrogel composition and environmental factors [176,177]. Concerns regarding the potential ecological consequences of hydrogel degradation products, particularly in aquatic environments, necessitate further research to ensure that these materials do not introduce harmful substances into ecosystems [178].

Economic considerations play a significant role in the practical application of stimulus-responsive hydrogels. The cost of raw materials, synthesis processes, and scalability of production can limit the widespread adoption of these materials in wastewater treatment [171,179]. Developing cost-effective synthesis methods and optimizing the use of readily available materials will be essential for making hydrogels a viable option for large-scale environmental applications.

Achieving high selectivity for specific pollutants remains a challenge for stimulus-responsive hydrogels. While these materials can effectively capture a range of contaminants, the presence of competing pollutants in wastewater can hinder their performance [180,181]. Additionally, maintaining long-term efficiency in pollutant removal is crucial for the practical application of hydrogels in dynamic wastewater environments. Ongoing research aimed at enhancing the selectivity and efficiency of hydrogels through tailored design and functionalization will be vital for overcoming these limitations [182,183].

**Figure 8:**
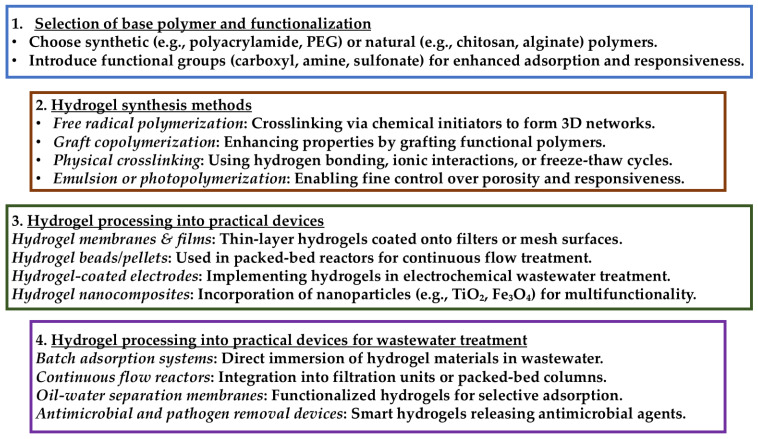
The practical applicability of stimulus-responsive hydrogels in wastewater treatment and environmental remediation.

To ensure the practical applicability of stimulus-responsive hydrogels in wastewater treatment and environmental remediation, a structured fabrication and processing approach is essential (Figure 8). The development of these hydrogels involves careful selection of base polymers, advanced synthesis techniques, and strategic functionalization to enhance their adsorption capacity and responsiveness. Furthermore, integrating these hydrogels into real-world devices requires optimized processing methods, such as membrane coatings, hydrogel beads, and nanocomposite formulations, to maximize efficiency and sustainability. The following section outlines the key fabrication steps and processing techniques necessary for the large-scale deployment of hydrogels in environmental applications.

To ensure the practical applicability of stimulus-responsive hydrogels in wastewater treatment and environmental remediation, a structured fabrication and processing approach is essential. The development of these hydrogels involves careful selection of base polymers, advanced synthesis techniques, and strategic functionalization to enhance their adsorption capacity and responsiveness. Furthermore, integrating these hydrogels into real-world devices requires optimized processing methods, such as membrane coatings, hydrogel beads, and nanocomposite formulations, to maximize efficiency and sustainability. The following section outlines the key fabrication steps and processing techniques necessary for the large-scale deployment of hydrogels in environmental applications.

## 6. Conclusions

Stimulus-responsive hydrogels demonstrate immense potential for addressing environmental pollution challenges, particularly in wastewater treatment. Their ability to adapt to external stimuli such as pH, temperature, light, and ionic strength enhances their efficiency in removing contaminants like heavy metals, dyes, and organic pollutants. The incorporation of nanomaterials and bio-based polymers further improves their structural properties, enabling scalable and sustainable environmental applications. Additionally, functional modifications allow for selective pollutant targeting and recyclability, supporting a circular economy approach.

Current trends in the field of responsive hydrogels are characterized by a growing emphasis on integrating advanced materials and technologies to enhance their performance. Researchers are increasingly exploring the use of smart nanocomposites that combine hydrogels with nanoparticles, which can improve the mechanical strength and responsiveness of the hydrogels while also offering additional functionalities such as photocatalytic degradation of pollutants. Furthermore, the trend towards bioengineering and the use of naturally derived polymers reflects a shift towards more environmentally friendly materials, which can reduce the ecological footprint of wastewater treatment processes.

Looking to the future, there is significant potential for the development of multifunctional hydrogels that not only treat wastewater but also monitor and detect contaminants in real time. The incorporation of sensor technology into hydrogels could lead to the creation of responsive systems that adjust their treatment capabilities based on the specific pollutants present, thus enhancing overall treatment efficiency. Moreover, advancements in 3D printing and other manufacturing techniques may enable the production of hydrogels with complex architectures tailored for specific applications, paving the way for innovative solutions in environmental remediation.

However, several challenges remain in the development and application of responsive hydrogels in wastewater treatment. One major concern is the long-term stability and durability of these materials in dynamic and harsh environmental conditions. Ensuring that hydrogels maintain their structural integrity and functionality over extended periods is crucial for practical applications. Additionally, the scalability of hydrogel production and their integration into existing wastewater treatment infrastructures pose significant hurdles. Researchers must navigate regulatory frameworks and economic considerations to facilitate the widespread adoption of these advanced materials.

In conclusion, while the promise of stimulus-responsive hydrogels in addressing environmental pollution is substantial, ongoing research and development efforts are essential to overcome existing challenges and realize their full potential. Future efforts should focus on optimizing hydrogel formulations for enhanced selectivity, long-term stability, and large-scale deployment, paving the way for cleaner water and sustainable environmental management. By addressing these challenges and leveraging current trends, we can move towards a more sustainable and effective approach to tackling the pressing issue of water pollution.

## Figures and Tables

**Table 1:** Common chemical groups or functionalities that are responsive to various stimuli, along with their structures.

Stimuli	Chemical Groups Functionalities	Structure	Reference
pH-responsive groups	Carboxylic acids (-COOH): these can lose a proton (H^+^) at higher pH values, making them responsive to pH changes.	O	[91,92]
		||	
		R-C-OH	
	Amino Groups (-NH_2_): these can gain a proton (H^+^) at lower pH values.	H	
		|	
		R-N-H	
		|	
		H	
Temperature-responsive groups	Poly(N-isopropylacrylamide) (PNIPAM): this is a polymer that exhibits a lower critical solution temperature (LCST) behavior.	O	[93]
		\ \	
		N—(CH(CH_3_)_2_)	
		(Repetitive unit)	
		//	
		O	
Light-responsive groups	Azobenzene (-N=N-): this group can undergo isomerization upon UV or visible light exposure.	R-S-S-R′	[94]
Redox-responsive groups	Disulfide bonds (-S-S-): these can be reduced to free thiols under reducing conditions.		[95]
Enzyme-responsive groups	Peptide bonds: these can be cleaved by specific proteolytic enzymes.	R1-C(=O)-N-R_2_	[96]
Magneto-responsive groups	Iron oxide nanoparticles: these can be influenced by magnetic fields.	Fe_3_O_4_ (simplified representation of iron oxide)	[97]
Chemical reaction-responsive groups	Chemistry functionalities (e.g., Azide-N_3_ and Alkyne -C≡C-): these can react under specific conditions to form stable compounds.	Structure of azide: N≡N-R Structure of alkyne: R-C≡C-R′	[98]
Hydrophobic/hydrophilic groups	Hydrophobic alkyl chains (R-): these can aggregate or disperse in response to solvent polarity.	CH_3_-(CH_2_)_n-_	[99]
Ionizable functional groups	Hydrophobic alkyl chains (R-): these can aggregate or disperse in response to solvent polarity.	O	[100]
		||	
		O-P-O^−^	
		|	
		O^−^	

**Table 2:** Applications of stimulus-responsive hydrogels in wastewater treatment.

The Specific Area of Wastewater Treatment Where the Hydrogel Is Applied.	Hydrogel Type	Mechanism of Action—The Hydrogel’s Method of Interacting with or Removing Pollutants.	Reference
Heavy metal ion removal	Polydopamine-modified clay hydrogel	Adsorption of heavy metal ions through complexation with ferric ions	[113]
	Chitosan/calcium alginate in a bentonite composite hydrogel	Ion exchange and adsorption of heavy metals	[114]
	Poly(sodium acrylate) hydrogel	pH-sensitive swelling for the removal of organic dyes	[115]
Pollutant removal	Biodegradable hydrogel from sugarcane bagasse	Swelling and hydrogen bonding with organic pollutants	[116]
	Cyclodextrin hydrogel	Adsorption of aromatic micropollutants through functional group interactions	[117]
	DNA-based hydrogel	Targeted delivery of antimicrobial agents	[118]
	Poly(ethylene glycol) (C_2n_H_4n+2_O_n+1_)-based hydrogel	Responsive release of antimicrobial agents	[119]
Oil and grease separation	Graphene hydrogel	Adsorption and separation of oil from water	[120]
Dye removal	ZnO/g-C_3_N_4_/Fe_3_O_4_ nanocomposite hydrogel	Photocatalytic degradation of organic contaminants	[121]
	Chitosan/acrylamide hydrogel	Antimicrobial action against bacteria and fungi	[122]

**Table 3:** Examples of stimulus-responsive hydrogels in controlling different types of pathogens.

Pathogen	Type	Hydrogel Mechanism of Action	Reference
Bacteria	*Escherichia coli*	Antimicrobial agents embedded in the hydrogel for wound dressings and food packaging	[155]
	*Staphylococcus aureus*	Swelling and trapping of bacteria for medical implants and catheters	[156]
Viruses	*Influenza virus*	Incorporation of antiviral agents for vaccine delivery and surface coatings	[157]
	SARS-CoV-2	Controlled release of antiviral agents for air filtration systems and masks	[158]
Fungi	*Candida albicans*	Antifungal compounds embedded in the hydrogel for topical treatments and implants	[159]
	*Aspergillus niger*	pH-responsive release of antifungal agents for agricultural applications	[158]
Parasites	*Plasmodium* spp.	Controlled release of antiparasitic drugs for drug delivery systems	[160]

## Data Availability

No new data were created or analyzed in this study. Data sharing is not applicable to this article.
